# Supplementary material for: Phenotypic and Molecular Characterization of Commensal, Community-Acquired and Nosocomial Klebsiella spp
Source: Microorganisms. 2021 Nov 12;9(11):2344. doi: 10.3390/microorganisms9112344 (PMC8625991; doi:10.3390/microorganisms9112344)
Supplement: Supplementary file 1 [file microorganisms-09-02344-s001.zip › microorganisms-1448813-supplementary.pdf]

Table S1. Oligonucleotides used in this study.

Name	Sequence (5′-3′)	Target Gene (s)	Reference
**Identification/Phylogeny**			
KrpoB-for	TTATGGACCAGAACAACC	*rpoB* (β subunit of bacterial RNA polymerase)	This study
KrpoB-rev	AACGGGATCAGGGC		
**Antibiotic Resistance**			
P1	ATGGTTAAAAAATCACTGCGCC	*blaCTX-M* (Extended-Spectrum β-lactamases)	24
P2b	TCCCGACGGCTTTCCGCCTT		
CTX-M-1G-F	AAAAATCACTGCGCCAGTTC	*blaCTX-M* (Extended-Spectrum β-lactamases)	23
CTX-M-1G-R	AGCTTATTCATCGCCACGTT		
CTX-M-2G-F	CGACGCTACCCCTGCTATT		
CTX-M-2G-R	CCAGCGTCAGATTTTTCAGG		
CTX-M-9G-F	CAAAGAGAGTGCAACGGATG		
CTX-M-9G-R	ATTGGAAAGCGTTCATCACC		
**Siderophores**			
iutA-F	GATGCCGCGACCATAGTATT	*iutA* (Aerobactin synthesis)	
iutA-R	TGAGTCGTTGCGATTCTACC		
iucB-F	TGTTCACCAACCAGCAGATG	*iutB* (Aerobactin receptor)	
iucB-R	GTTCACAGCGGATATGGACT		
fepA-F	TACTTCTTCGGCTCCTGCTT	*fepA* (Enterobactin synthesis)	This study
fepA-R	CAGCAACCAGTTCACGGATA		
fepC-F	CTTGTTCACCGCCTCTTCAT	*fepC* (Enterobactin receptor)	
fepC-R	TCACTTCACCGCCATTATCG		
FyuA-F	CCTTCCGGTTCGTTAATCGT	iutA (Yersibactin synthesis)	
FyuA-R	GCCATAGCGCGTATCAATCA		
YbtT-F	GTGACGCAATCTGCAATGTG	iutB (Yersibactin receptor)	
YbtT-R	ATCCGCCAATGTCTATCAGC		
**Virulence factors**			
magA-F	GGTGCTCTTTACATCATTGC	*magA* (hypermucoviscosity phenotype)	26
magA-R	GCAATGGCCATTTGCGTTAG		
rmpA-F	ACTGGGCTACCTCTGCTTCA	*rmpA* (hypermucoviscosity phenotype)	27
rmpA-R	CTTGCATGAGCCATCTTTCA		
wabG-F	ACCATCGGCCATTTGATAGA	*wabG* (lipopolysaccharide synthesis)	
wabG-R	CGGGGCAGATCCATATC		
uge-F	TCTTCACGCCTTCCTTCACT	*uge* (uridine diphosphate galacturonate 4-epimerase)	
uge-R	GATCATCCGGTCTCCCTGTA		
kfuB-F1179	GAAGTGACGCTGTTTCTGGC	*kfu* (iron-uptake system)	
kfuC-R649	TTTCGTGTGGCCAGTGACTC		
fimH-F	TGCTGCTGGGCTGGTCGATG	*fimH* (type 1 fimbrial adhesin)	
fimH-R	GGGAGGGTGACGGTGACATC		

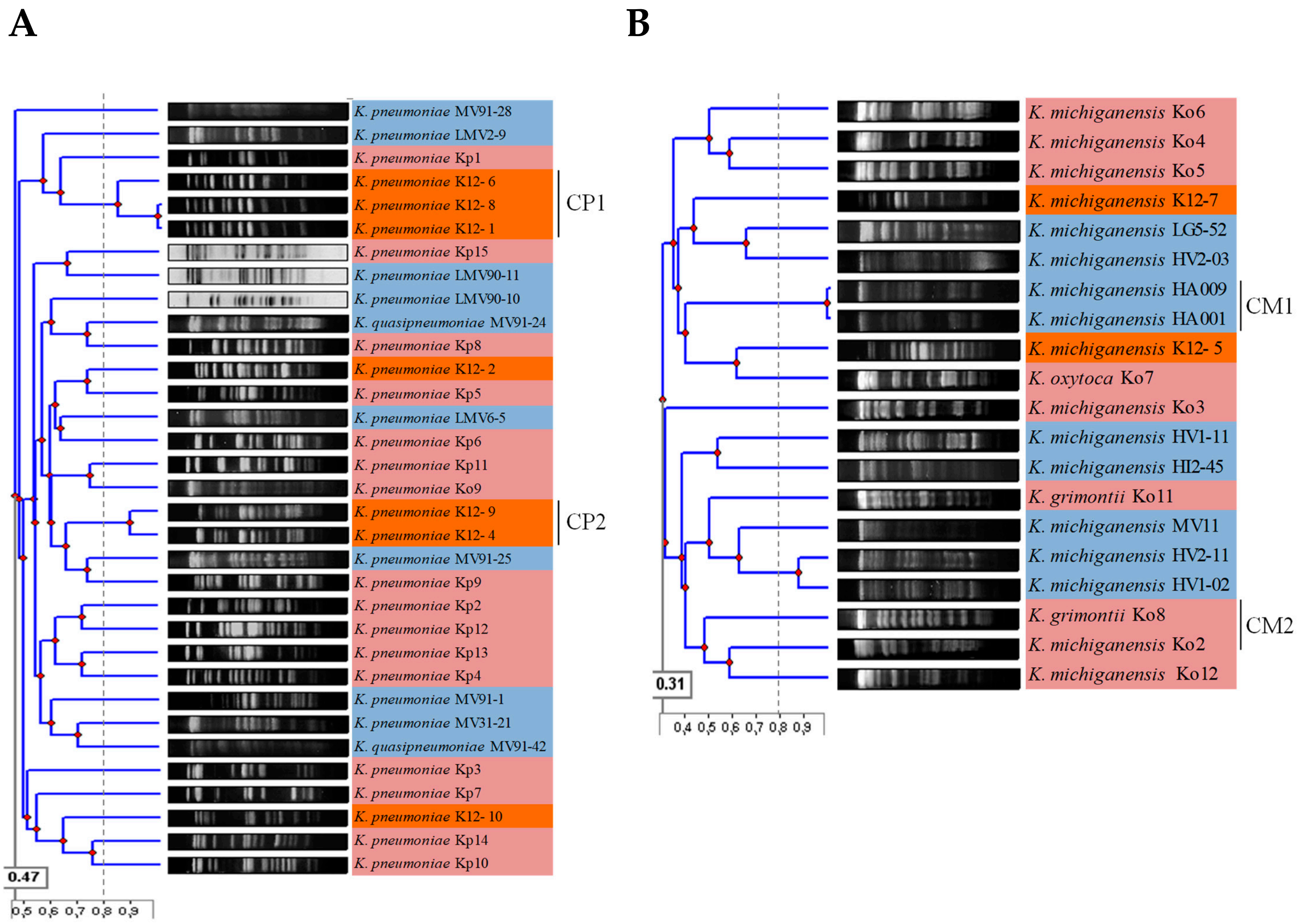

Figure S1. PFGE profiles of the isolates from the *K. pneumoniae* (A) and *K. oxytoca* complex (B).

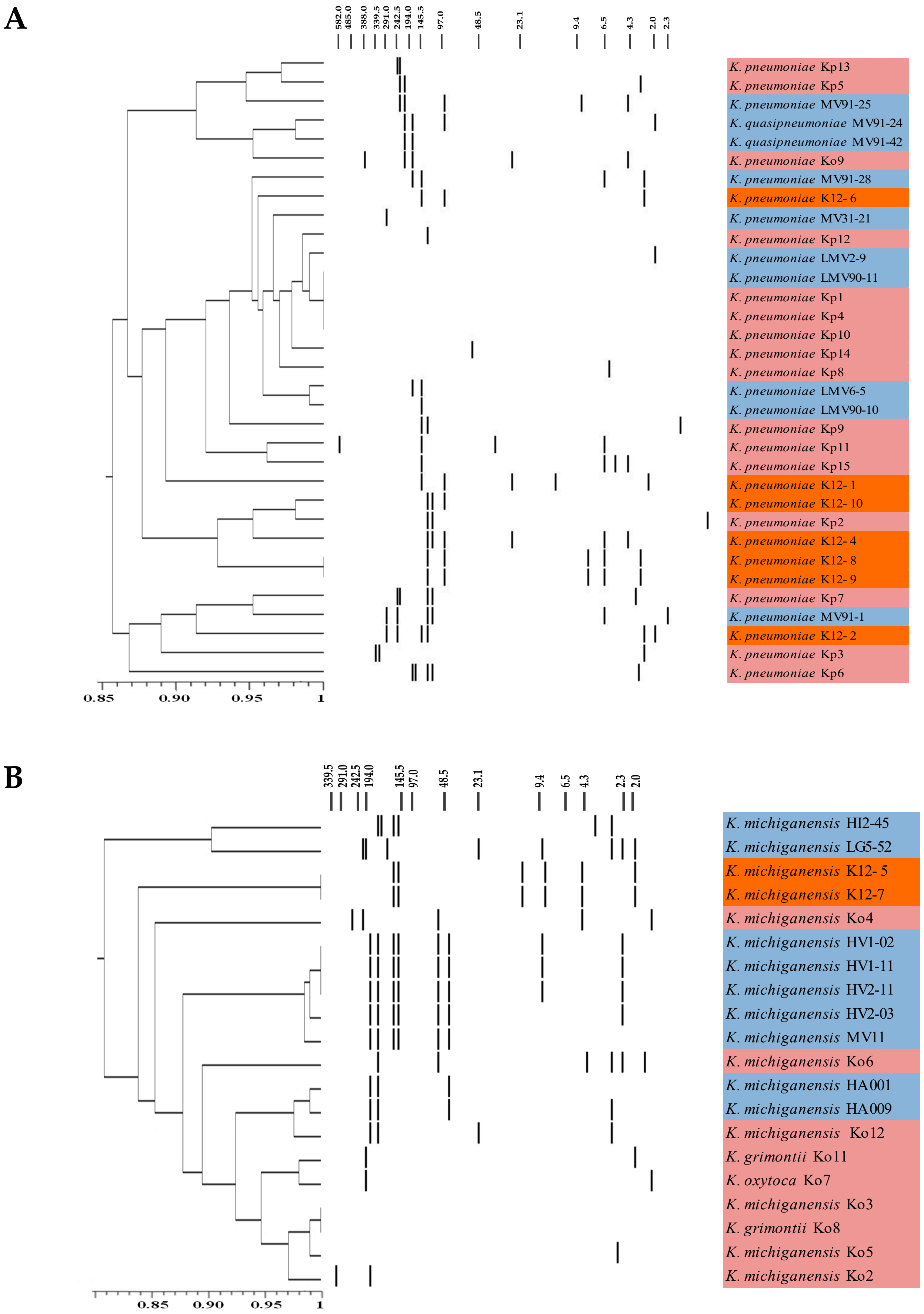

Figure S2. Plasmid profiles of isolates from *K. pneumoniae* (A) and *K. oxytoca* complex.
